# Next-generation DNA sequencing of *HEXA*: a step in the right direction for carrier screening

**DOI:** 10.1002/mgg3.37

**Published:** 2013-09-16

**Authors:** Jodi D Hoffman, Valerie Greger, Erin T Strovel, Miriam G Blitzer, Mark A Umbarger, Caleb Kennedy, Brian Bishop, Patrick Saunders, Gregory J Porreca, Jaclyn Schienda, Jocelyn Davie, Stephanie Hallam, Charles Towne

**Affiliations:** 1 Division of Genetics, Department of Pediatrics, Floating Hospital for Children, Tufts Medical CenterBoston, Massachusetts; 2 Good Start Genetics Inc.Cambridge, Massachusetts; 3 Division of Genetics, Department of Pediatrics, University of MD School of MedicineBaltimore, Maryland; 4 Dana Farber Cancer InstituteBoston, Massachusetts

**Keywords:** Ethnic-based screening, genetic screening, hexosaminidase A, next-generation DNA sequencing, Tay-Sachs disease.

## Abstract

Tay-Sachs disease (TSD) is the prototype for ethnic-based carrier screening, with a carrier rate of ∼1/27 in Ashkenazi Jews and French Canadians. HexA enzyme analysis is the current gold standard for TSD carrier screening (detection rate ∼98%), but has technical limitations. We compared DNA analysis by next-generation DNA sequencing (NGS) plus an assay for the 7.6 kb deletion to enzyme analysis for TSD carrier screening using 74 samples collected from participants at a TSD family conference. Fifty-one of 74 participants had positive enzyme results (46 carriers, five late-onset Tay-Sachs [LOTS]), 16 had negative, and seven had inconclusive results. NGS + 7.6 kb del screening of *HEXA* found a pathogenic mutation, pseudoallele, or variant of unknown significance (VUS) in 100% of the enzyme-positive or obligate carrier/enzyme-inconclusive samples. NGS detected the B1 allele in two enzyme-negative obligate carriers. Our data indicate that NGS can be used as a TSD clinical carrier screening tool. We demonstrate that NGS can be superior in detecting TSD carriers compared to traditional enzyme and genotyping methodologies, which are limited by false-positive and false-negative results and ethnically focused, limited mutation panels, respectively, but is not ready for sole use due to lack of information regarding some VUS.

## Introduction

Tay-Sachs disease (TSD) is an autosomal recessive neurodegenerative disease that has served as the prototype for ethnic-based screening programs throughout the world. Screening for TSD in people of Ashkenazi Jewish (AJ) (eastern European) descent has been almost universally accepted as important for preconception/prenatal care. With an approximately one in 27 carrier rate (Scott et al. 2010), use of a highly sensitive methodology is critical for screening. In addition, high carrier rates in other populations, such as the French Canadian, Irish, and Cajun, have underscored the need for a method that can be used to screen an ethnically diverse population accurately.

In the early 1970s, Jewish communities designed screening programs for the detection of TSD carrier status (Kaback et al. 1993). The initial TSD screening programs were very successful in providing Jewish individuals and couples with information to allow for the birth of unaffected offspring through reproductive technologies or mate selection. By 1992, almost one million individuals had been screened and the number of children born with TSD to AJs had decreased from ∼45 to 3–4 per year, lower than the incidence in the non-Jewish population (Kaback et al. 1993).

While the AJ population still has one of the highest carrier rates for TSD, TSD carrier rates are elevated in other populations as well; the carrier frequency is one in 25 in those of French Canadian descent (Andermann et al. 1977), up to one in 52 people of Irish descent (van Bael et al. 1996; Branda et al. 2004), and there is an increased carrier frequency in Cajuns (McDowell et al. 1992). Based on incidence in the general population, the TSD carrier rate is calculated to be one in 300, with one in 170 found to be carriers (many due to a pseudodeficiency allele) on prospective enzyme studies (Triggs-Raine et al. 1992). As the intermarriage rate of people of AJ descent and other ethnicities increases, screening has become more complex and an ethnicity-independent approach has become increasingly important. Recently, a study of the carrier frequencies of more than 20,000 ethnically diverse patients has provided definitive support for a pan-ethnic screening approach (Lazarin et al. 2013).

The original screening programs assayed hexosaminidase A (HexA, encoded by the gene *HEXA*, OMIM accession number *606869) activity to determine carrier status. This type of “traditional” enzyme analysis has long been considered the gold standard for TSD carrier screening, detecting approximately 98% of carriers from all ethnic backgrounds (Natowicz and Prence 1996; Monaghan et al. 2008). Carriers are classified as those individuals with enzymatic activity levels in the ∼36–52% range, while noncarriers have ∼60–73% enzyme activity, and Sandhoff patients typically measure within the ∼76–85% range (but this is evaluated in the context of the total Hex activity as well), and values may vary between laboratories. However, the HexA enzyme assay has some limitations. It specifically requires a blood sample, and the enzyme activity can be affected by several factors including pregnancy and certain medications such as oral contraceptives (D'Souza et al. 2000) and antihypertensives. There is also an inconclusive range in which it is difficult to clearly distinguish TSD carriers from noncarriers. Furthermore, enzyme analysis can produce false negatives and false positives due to the presence of B1 and pseudodeficiency alleles, respectively. The B1 variant renders the protein capable of hydrolyzing some artificial substrates commonly used in screening assays to measure HexA activity, yielding HexA activity within or near the normal range. However, the B1 variant is unable to hydrolyze its natural GM2 ganglioside substrate in vivo*,* and thus is a pathogenic variant. In contrast, a *HEXA* pseudodeficiency variant is a nonpathogenic variant, yet its presence results in reduced enzymatic activity in vitro. These types of limitations with enzyme analysis have driven the TSD carrier screening community toward the development of DNA-based assays.

Several types of DNA analysis can be used to identify TSD carriers successfully. Currently, the most widely used methodology is a targeted genotyping approach. This is typically performed using a DNA screening panel that consists of a limited set of mutations, as opposed to full-length target gene sequencing. For example, TSD carrier frequency data reported by Lazarin et al. (2013), used high-throughput genotyping to determine if 11 specific *HEXA* sequence variations were present. Although this type of analysis has its benefits, only those mutations commonly seen in the AJ population are included in most DNA screening panels, which generally yields a detection rate of ∼89–98% for AJs (Kaback et al. 1993; Schneider et al. 2009), but less than 60% for non-AJs (Kaback et al. 1993), and between 60 and 90% for those of mixed descent. In certain countries, DNA mutation screening may be the sole method used due to homogeneous Jewish populations or added cost of additional enzyme analysis. Several authors suggest that DNA mutation scanning should replace enzyme screening based on the possibility that DNA testing detects fewer false positives and inconclusive results (DeMarchi et al. 1996; Bach et al. 2001). However, due to the increasing rate of intermarriage in America, DNA screening for people who report AJ descent has become less sensitive. The current protocol for TSD carrier screening recommends that both enzyme and DNA analysis be performed in all cases, to provide the highest level of sensitivity and information for reproductive planning (National Tay-Sachs and Allied Diseases [NTSAD] Policy statement, [Schneider et al. 2009]). The success of targeted genotype screening in the AJ community was aided by the fact that three known *HEXA* mutations account for the vast majority (up to 98%) of TSD disease alleles in this group. In most other populations the molecular basis for TSD appears to be highly heterogeneous. While population-specific variants have been identified for some other ethnic groups, such as the 7.6 kb deletion for French Canadians, or c.459+5G>A for Spaniards, they are less predominant and make up a smaller fraction of disease alleles.

Full-length *HEXA* gene sequencing has the potential to replace targeted genotyping methodologies and enzyme analysis for TSD carrier screening if it can be used in a highly accurate manner. Extensive *HEXA* sequencing would be expected to diagnose nearly all carriers, including novel frame-shift, splice-site, and truncating variants, and is not subject to ethnicity limitations. Sanger sequencing has historically been considered to be the most accurate sequencing method, yet it is cost-prohibitive for use as a clinical carrier screening tool. In contrast to Sanger sequencing, next-generation DNA sequencing (NGS) has the potential to enable the sequencing of the entire *HEXA* gene in a more cost-effective manner. We recently described an NGS-based approach for the assessment of carrier status with respect to a panel of genetic disorders including TSD (Umbarger et al. 2013). Here, we use this highly optimized NGS technology to sequence the *HEXA* exons and exon–intron junctions to assess whether this approach would yield detection rate, false-positive rate, and false-negative rate equivalent or better than that achieved with traditional enzyme analysis and/or targeted genotyping. Such information may be useful in determining the current, optimal screening protocol for Tay-Sachs disease.

## Material and Methods

### Patients

DNA samples were collected from 74 individuals attending the National Tay-Sachs and Allied Diseases Annual Family Conference in Boston on 31 March–3 April 2011 in order to obtain samples with a wide range of Tay-Sachs disease mutations. Approximately 80% of the families who attended had a family history of TSD, thus, unlike in population screening studies, carrier status was known for many of the individuals enrolled. After Tufts University School of Medical Institutional Review Board approved informed verbal consent was obtained from each participant, peripheral blood samples were collected for both TSD enzyme analysis and NGS (plus 7.6 kb del) analysis. Samples were coded by number to link blood samples for enzyme and DNA analysis and the participant's questionnaire regarding age, ethnic background, family and personal history of genetic diseases, previously determined carrier status, gender, pregnancy status, medication use, as well as medical history for bone marrow transplant, liver disease, and hypertension.

### HexA enzyme analysis

Serum from the blood samples was separated, frozen, and sent to the University of Maryland Biochemical Genetics Laboratory for testing. To determine TSD carrier status, serum HexA activity was assayed in 74 samples according to the standard heat inactivation method using an artificial fluorogenic substrate described previously (Ben-Yoseph et al. 1985). Reference ranges were those established in the testing laboratory (carriers ∼36–52%, noncarriers ∼60–73%, Sandhoff ∼76–85% with total HEX activity taken into account). Given that serum HexA values are affected by a number of factors (O'Brien et al. 1970), interpretation of results took into consideration gender, pregnancy status, bone marrow status, and use of medications for liver disease, hypertension, and oral contraceptives. Only serum samples were available for testing.

### DNA analysis

DNA extraction was performed to yield highly purified genomic DNA suitable for downstream molecular biology steps, such as polymerase chain reaction (PCR). The genomic DNA was utilized in a process that selectively captures only genomic DNA sequences that are of clinical interest (Umbarger et al. 2013). The captured DNA sequences were amplified via PCR, and then sequenced using the Illumina HiSeq (Illumina, San Diego, CA). The resulting raw data were analyzed by a bioinformatics pipeline that reports variants in the processed genotype calls (Umbarger et al. 2013). All detected variants were confirmed by Sanger sequencing on an ABI 3730xl. AmpliTaq Gold (Applied Biosystems, Carlsbad, CA) was used to amplify regions of interest containing the position to be confirmed. PCR and BigDye terminator Cycle Sequencing version 3.1 master mix (Applied Biosystems, Foster City, CA) was used to generate fluorescently labeled products that could be detected by the 3730xl (Applied Biosystems, Carlsbad, CA). ABI data was analyzed by Mutation Surveyor v4.0 (Softgenetics, State College, PA). A three-primer PCR protocol was used to screen for patients with the common 7.6 kb deletion in *HEXA*. HX-7.6 kb R1 5′-AGC CTG GGC AAC ACA GTA AG-3′ (common primer) lies on the 3′ side of the deleted region, and therefore amplifies both the wild-type (WT) and deletion alleles. HX-7.6 kb (WT) F2 5′- GGC TTA CAG CAA CCT CGA AC 3′ lies within the deleted region and amplifies only the WT allele. HX-7.6 kb del F2 5′-TGG GGT ACC TGA GAT GTT TTG-3′ lies outside the deleted region on the 5′ side, and generates a PCR product only when it is in proximity to the HX-7.6 kb R1 primer, due to the presence of the deletion. After PCR, the products were used in a SNaPshot (Applied Biosystems, Carlsbad, CA) genotyping reaction that was run on an ABI 3730xl (Applied Biosystems, Carlsbad, CA), and analyzed with GeneMapper v1.95 (Applied Biosystems, Foster City, CA). *HEXA* GenBank accession number NM_000520.4

## Results

### Characteristics of study participants

**Figure 1:**
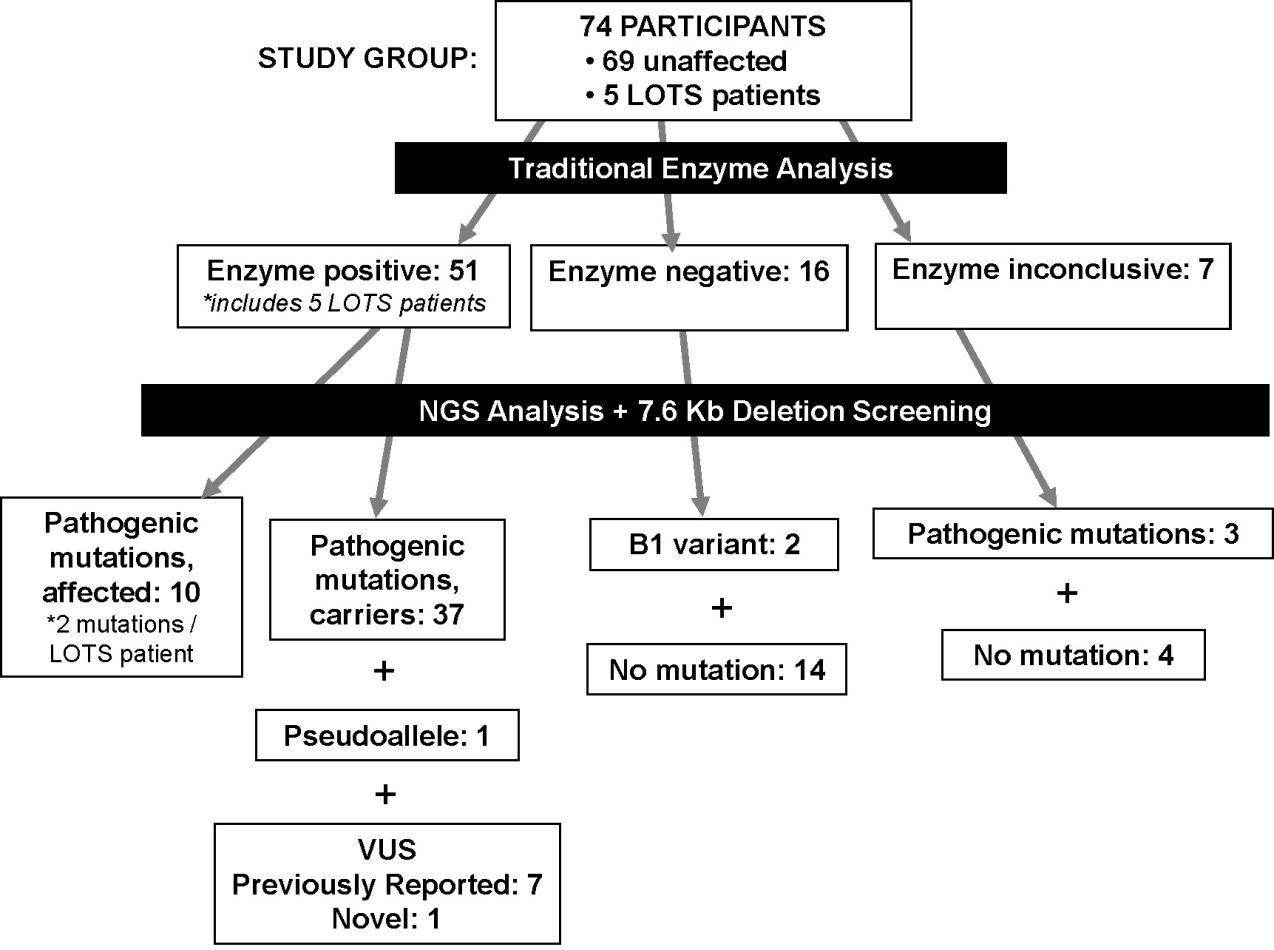
The study design and outcome. Enzyme analysis and DNA analysis results are shown for all study participants. The white boxes show the number of positives, negatives, inconclusives, and the number of alleles for each mutation category. Note that a pseudoallele results in a false positive in the enzyme analysis, while a B1 variant produces a false-negative result. VUS, variant of unknown significance.

A total of 74 adult individuals were recruited for the study (Fig. 1). Some participants may have been blood relatives. Five participants reported being affected with late-onset Tay-Sachs disease (LOTS); 36 participants reported themselves as obligate carriers, 11 as carriers for TSD, eight as noncarriers, and one as a carrier for a pseudodeficiency allele. Two participants reported themselves as carriers for Sandhoff disease. Eleven participants did not respond to the question about TSD carrier status.

Of the total participants, 27 individuals, including three with LOTS, reported themselves to be at least partially of AJ descent, two were African-American or African-American/Native American, four Hispanic or Hispanic with European admixture, one Indian and one Native American, both with Caucasian admixture. The other participants were Caucasian.

### Enzyme and DNA analysis results

An overview of the study group, and a summary of the enzyme and DNA analysis results, is shown in Figure 1. Specific variants found are noted in Tables 1, 2, 3, and their corresponding enzyme levels are shown in Table S1. All LOTS participants had enzyme results indicative of affected status. Two mutations were detected in all samples from affected participants, and all carried one copy of the mild p.Gly269Ser (c.805G>A) allele, as might be expected for LOTS participants. Of the remaining 69 samples from unaffected individuals, enzyme analysis classified 46 as carriers, 16 as noncarriers, and seven as inconclusive.

**Table 1:** Mutations observed in this study

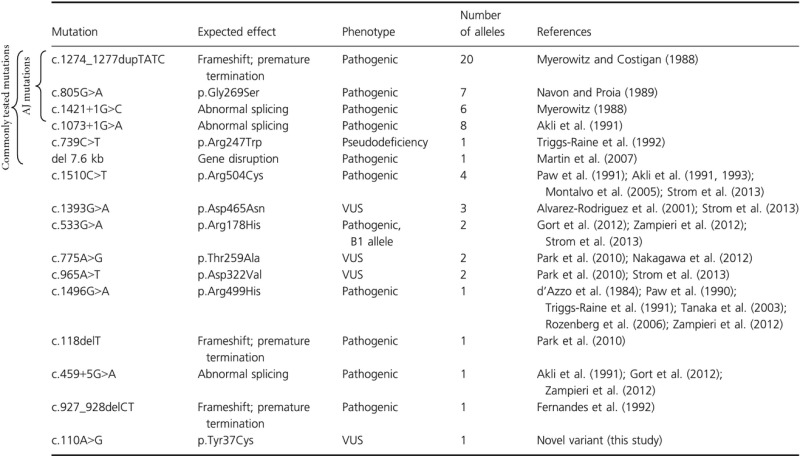

**Table 2:** Analysis of the functional impact of missense variants of unknown significance (VUS) by three computational methods

Variant	Polyphen2, HumVar set (score)	Sift (score)	Mutation assessor functional impact (score)	Variant category and reference
p.Tyr37Cys	Benign (0.296)	Tolerated (0.09)	Low (1.4)	Novel variant (this study)
p.Thr259Ala	Possibly damaging (0.903)	Tolerated (0.36)	Low (1.4)	Previously reported (Park et al. 2010; Nakagawa et al. 2012)
p.Asp465Asn	Probably damaging (0.990)	Tolerated (0.22)	Medium (2.8)	Previously reported (Alvarez-Rodriguez et al. 2001; Strom et al. 2013)
p.Asp322Val	Probably damaging (0.999)	Damaging (0)	High (4.3)	Previously reported (Park et al. 2010; Strom et al. 2013)

**Table 3:** Mutation status of samples with inconclusive enzyme results

Study number	Self-reported TS status	Enzyme result	*HEXA* sequence analysis
54	Obligate carrier (parent)	Inconclusive	c.459+5G>A
51	Obligate carrier (parent)	Inconclusive	p.Arg499His
24	Obligate carrier (parent)	Inconclusive	c.l274_1277dupTATC
67	Not reported	Inconclusive	No change
*66*	Not reported	Inconclusive	No change
65	Not reported	Inconclusive	No change
57	Not reported	Inconclusive	No change

The 7.6 kb deletion common in French Canadians was found in one individual by genotyping and NGS detected known mutations or potentially pathogenic sequence changes in 51 samples from unaffected participants. No such changes were found by either DNA analysis method in 18 samples (Fig. 1). NGS also detected the following benign changes: the two common variants c.1518A>G (rs4777502) and c.1306A>G (rs1800431) were present in all samples, c.1195A>G (rs1800430) was found in an African-American noncarrier, c.9C>T (rs1800428) was present in two carriers of c.1073+1G>A, and a synonymous variant c.1338T>C (rs34085965) was found in the 7.6 kb deletion carrier. No sample included both a VUS and pathogenic mutation.

### Mutation distribution

A total of 16 unique mutations were observed (Table 1). Three mutations prevalent in the AJ population accounted for more than half (33/61) of identified TSD alleles (Table 1). Three additional common pathogenic or pseudodeficiency variants that are part of most mutation panels used in targeted mutation analysis (according to GeneTests) were detected in another 10 alleles. In addition, 10 variants, one of them novel, represented the remaining 29% (18/61) alleles.

The missense mutation p.Arg504Cys was found in two carriers and two LOTS individuals of diverse Caucasian backgrounds; this mutation previously has been reported as pathogenic in several populations (Akli et al. 1991, 1993; Montalvo et al. 2005). Functional studies demonstrated that the mutant protein has no enzymatic activity (Paw et al. 1991). Also, c.459+5G>A, a common disease allele in Spanish and Argentinean patients, was seen in one Hispanic individual (Akli et al. 1991; Gort et al. 2012; Zampieri et al. 2012). Arg499His was found in one parent of a patient with juvenile TSD. This mutation has been previously associated with juvenile or subacute disease (Paw et al. 1990; Tanaka et al. 2003; Rozenberg et al. 2006; Zampieri et al. 2012). c.927_928delCT (Fernandes et al. 1992) and c.118delT (Park et al. 2010) appear to be rare alleles; they are considered pathogenic because of their truncating nature. Of note, these five variants would not have been detected by utilizing popular genotyping approaches, like the one described in Lazarin et al. (2013) because they are not among the 11 *HEXA* variants on their panel.

The rare missense mutations p.Asp465Asn, p.Thr259Ala, p.Asp322Val, and p.Tyr37Cys were classified as variants of unknown significance (VUS). No functional studies are available. We performed sequence-based functional predictions using three different tools (PolyPhen2 [Adzhubei et al. 2010], SIFT [Kumar et al. 2009], and MutationAssessor [Reva et al. 2011]; Table 2) which yielded concordant classifications of high functional impact for p.Asp322Val, and low functional impact for p.Tyr37Cys. The methods yielded discordant classifications for p.Thr259Ala and p.Asp465Asn. Recent speculation based on crystallographic modeling of p.Thr259Ala indicates that this mutation is likely to be detrimental due to its key location in the catalytic core domain. It is proposed that a mutation of Thr to Ala at this position would remove important stabilizing hydrogen bonds and thus disrupt the overall catalytic domain structure (Nakagawa et al. 2012). However, definite evidence of the enzymatic effects of substitutions at this residue awaits in vitro functional studies. p.Asp465Asn was previously found in homozygous state in a patient with TSD and several members of his extended family that were enzymatically defined as TSD carriers (Alvarez-Rodriguez et al. 2001). It was again observed in three carriers in our study, although there is some indication, based on demographic data provided, that two of the individuals may be related. Both p.Thr259Ala and p.Asp322Val were found twice in enzyme positive, reported (obligate) carriers in this study. It is again likely that the participants carrying the same mutation are related. We might assume that they are identical to the individuals in whom the two mutations were characterized for the first time by Park et al. (2010), as the Park study drew from the same family support group as our study. The p.Tyr37Cys mutation is novel and was observed in an enzyme-positive Polish individual who did not report a family history of TSD.

### Comparison of study methods

Enzyme analysis demonstrated carrier status for 42 of the 47 self-reported carriers, 36 of whom are obligate carriers. For three additional obligate carriers, the enzyme results were inconclusive; those individuals were found to be carriers of known pathogenic mutations by DNA analysis (Table 3). Two obligate carriers with a B1 allele (p.Arg178His) were classified as noncarriers by enzyme analysis. False-negative results are expected with the B1 allele, as discussed previously.

NGS supplemented by genotyping for the 7.6 kb deletion found sequence changes in all 47 self-reported carriers, although in seven cases the sequence changes are considered to be VUS. All seven of those samples were positive by enzyme analysis. Three individuals with unspecified carrier status were identified as carriers by enzyme analysis; sequence analysis detected two common mutations and a novel VUS (p.Tyr37Cys) in these participants.

Sensitivity for enzyme analysis of serum samples in this study was 89% (42/47 self-reported carriers); sensitivity for DNA analysis (counting the seven samples with VUS as false negatives) was 85% (40/47); however, if samples with VUS were included as true positives, DNA sensitivity rose to 100%. Only 68% (32/47) of the self-reported carriers had mutations that are commonly tested for in most commercial screening panels, or 72% (34/47) for the extended panel described by Lazarin et al. (2013). Ninety-one percent (43/47) of carriers were either enzyme positive or had a commonly tested mutation. Enzyme analysis combined with NGS and 7.6 kb deletion analysis identified all known carriers in this study.

Enzyme analysis, as would be expected, incorrectly assigned carrier status to the individual with a pseudodeficiency allele, did not detect two B1 alleles, and was inconclusive in four individuals with unknown carrier status, who tested negative by DNA analysis.

## Discussion

Full gene sequencing of *HEXA* using a NGS platform has the ability to provide additional information regarding TSD carrier status when compared to traditional enzyme analysis and targeted genotyping approaches. This type of analysis will not generate false-positive and false-negative results due to the presence of pseudodeficiency or B1 alleles, respectively, as enzyme analysis does. Also, NGS provides added diagnostic value over limited mutation panels because it is an ethnicity-independent method, and it can detect novel variants. For example, the high-throughput genotyping panel described by Lazarin et al. (2013) tests for 11 *HEXA* variants. If such a panel alone had been used on our study population, then 11 carriers would have gone undetected. Our sample is skewed in that it comes from a population of relatives of those born with Tay-Sachs, reflecting those populations that are currently less often offered screening for Tay-Sachs due to ethnic background.

Our data clearly show that the use of a limited genotyping panel based on ethnicity would not be expected to detect all TSD carriers in a pan-ethnic population. Most genotyping panels are based on current American College of Obstetricians and Gynecologists/American College of Medical Genetics (ACOG/ACMG) screening recommendations and they tend to work well only in limited populations, such as the AJ (Lazarin et al. 2013). Full gene sequencing is superior over limited genotyping panels for the detection of TSD carriers in other ethnic groups because data are limited as to the predominant *HEXA* variants in different populations, thus these variants would not be included in typical genotyping panels. However, for NGS clinical carrier screening to become a widely accepted stand-alone carrier screening tool, more work needs to be done to assign pathogenicity status to newly discovered or rare variants. Specific advantages and limitations of the NGS technology used in this study in relation to the currently used clinical carrier testing methods are discussed in more detail below.

Our results suggest that combining enzyme analysis with NGS (plus genotyping for 7.6 kb) may be sufficient to detect all TSD carriers, but neither method is adequate currently to detect all variants as a stand-alone technology. Enzyme analysis has the higher sensitivity in our study, at least when samples with VUS are counted as false negatives for DNA analysis. No leukocyte samples were available for analysis, which would likely have yielded a lower inconclusive rate than serum analysis for HexA. Enzyme analysis does not detect carriers of B1 alleles. p.Arg178His, the most frequent B1 allele, has been reported in up to 20% of disease alleles in Spanish, Italian, Argentinian, and Brazilian TSD patients (Montalvo et al. 2005; Rozenberg et al. 2006; Gort et al. 2012; Zampieri et al. 2012); thus, screening by enzyme analysis alone will miss a significant fraction of carriers in certain ethnic groups (i.e., false negatives). In addition, the relatively high frequency (32%) of the pseudodeficiency allele p.Arg247Trp in non-Jewish, enzyme-positive individuals (Triggs-Raine et al. 1992) requires follow-up testing with a DNA-based method to clarify carrier status (results summary shown in Fig. 1).

With the exception of the individual with the large 7.6 kb deletion common in French Canadians, NGS detected a nucleotide change in all presumptive carriers, although it remains unclear whether all are the causative mutations. Most importantly, NGS identified a number of mutations that are not used for targeted carrier screening, but may be important in non-Jewish populations. Examples of such mutations detected in our cohort are p.Arg504Cys, which may represent a relatively common European disease allele, c.459+5G>A, recently shown to be the most prevalent mutation in Spanish and Argentinean populations (Akli et al. 1991; Gort et al. 2012; Zampieri et al. 2012), and p.Arg499His, a mutation identified in such diverse ethnic backgrounds as Japanese (Tanaka et al. 2003) and Brazilian (Rozenberg et al. 2006).

NGS of *HEXA* also detected a number of rare missense variants. These variants were classified as VUS, as prevalence data were not sufficient and experimental data were not available to establish or exclude pathogenicity; computational methods commonly used for research purposes have not yet reached the sensitivity and specificity required for clinical applications. As only exons (plus short adjacent intronic stretches) were sequenced, deep intronic or promoter mutations are missed. Similarly, large deletions other than the known, common 7.6 kb deletion were not detected in this study. In general the literature indicates the frequency of intronic and large structural *HEXA* variants involved in TSD is believed to be very low; however, this may be an underestimate caused by ascertainment bias. Full-exon sequencing is not always sensitive enough to detect every disease allele in TSD patients or obligate carriers (Park et al. 2010; Gort et al. 2012). It is therefore conceivable that we did not detect the true disease-causing variants in at least some of the cases.

Overall, the results of our study indicate that TSD carrier screening using enzyme analysis combined with DNA analysis provides the highest level of sensitivity and information for reproductive planning. It is important to emphasize that for individuals who are not of AJ descent or who are uncertain about their ancestry, targeted mutations screening (as currently provided by most labs) is not sufficient to identify all relevant disease alleles. Even in AJs, targeted mutation analyses will miss 5–10% of all carriers. Hence, NGS coupled with enzyme analysis, represents the most accurate approach.

Full gene sequencing in large screening population samples is expected to uncover many variants, some of them known to be benign, some rare, some not yet described. As our sample was intentionally highly skewed toward TSD carriers in order to assay as many mutations as possible, we cannot assess how many VUS (and potential false positives) are expected to be observed in the general population. This creates a challenge for clinical laboratories in determining which sequence variants should be reported out as pathogenic, and may in fact complicate matters unnecessarily. Unlike in diagnostic settings, reporting of VUS in population carrier screens does not seem appropriate, in particular if they do not add any real value to a decision making process due to the lack of proof of pathogenicity. NGS allows the inclusion of as many variants as desired at the same cost; as new information becomes available, the list of pathogenic variants can be increased without the need to redesign assays. Although the current cost of NGS sequencing reagents are roughly comparable to that of genotyping, capital costs may be substantially different, and so the costs are hard to pinpoint (Umbarger et al. 2013).

To date, our experience with NGS as a method for carrier detection suggests that sequencing should still be complemented by enzyme analysis. However, based on a similar study of National Tay-Sachs and Allied Diseases Annual Family Conference participants Strom et al. suggested that *HEXA* sequencing combined with 7.6 kb del analysis could replace the enzyme assay in a non-AJ population (Strom et al. 2013). Our data clearly show that NGS is superior over the commonly used limited gene panels because of its ability to detect rare and novel variants. As we gain more insight from the data that become available, and improve classification methods for rare variants, a future is plausible where HexA enzyme testing is rendered entirely unnecessary.

## Supporting Information

**Table S1.** Data for individual study participants, including self-reported TS status, enzyme levels and interpretation, HexA variant(s) name(s) (if detected), and self-reported ethnicity.
