# Supplementary material for: Next-generation DNA sequencing of HEXA: a step in the right direction for carrier screening
Source: Mol Genet Genomic Med. 2013 Sep 16;1(4):260–8. doi: 10.1002/mgg3.37 (PMC3865593; doi:10.1002/mgg3.37)
Supplement: Supplementary file 1 [file mgg30001-0260-SD1.pdf]

Supp. Table S1. Data for individual study participants, including self-reported TS status, enzyme levels and interpretation, HexA variant(s) name(s) (if detected), and self-reported ethnicity

Patient number:	Self-reported TS status:	% Hex A activity:	Enzyme interpretation:	HexA variant(s) names, cDNA and protein, if applicable:	Self-reported ethnicity:
1	Affected - LOTS	8.6	Affected - LOTS	c.805G>A (p.Gly269Ser); c.1274_1277dupTATC	Ashkenazi Jewish
2	Affected - LOTS	7.5	Affected - LOTS	c.805G>A (p.Gly269Ser); c.1274_1277dupTATC	German/Russian
3	Affected - LOTS	6.2	Affected - LOTS	c.805G>A (p.Gly269Ser); c.1421+1G>C	Ashkenazi Jewish
4	Affected - LOTS	12.7	Affected - LOTS	c.805G>A (p.Gly269Ser); c.1510C>T (p.Arg504Cys)	Polish/Dutch/Old English
5	Affected - LOTS	11.3	Affected - LOTS	c.805G>A (p.Gly269Ser); c.1510C>T (p.Arg504Cys)	Ashkenazi Jewish/German
6	Carrier	46.5	Carrier	c.1274_1277dupTATC	Ashkenazi Jewish
7	Carrier	46.1	Carrier	c.1274_1277dupTATC	Ashkenazi Jewish
8	Carrier	46.9	Carrier	c.1274_1277dupTATC	Ashkenazi Jewish
9	Carrier	46.8	Carrier	c.1274_1277dupTATC	Ashkenazi Jewish
10	Carrier	25.9	Carrier	c.1274_1277dupTATC	Ashkenazi Jewish
11	Carrier	36.7	Carrier	c.1274_1277dupTATC	Ashkenazi Jewish
12	Not reported	47.5	Carrier	c.1274_1277dupTATC	Czechoslovakian
13	Carrier (obligate)	51.7	Carrier	c.1274_1277dupTATC	Ashkenazi Jewish/French Canadian/Ukrainian
14	Carrier (obligate)	33.3	Carrier	c.1274_1277dupTATC	Ashkenazi Jewish/Caucasian
15	Carrier (obligate)	46.7	Carrier	c.1274_1277dupTATC	Caucasian
16	Carrier (obligate)	41.4	Carrier	c.1274_1277dupTATC	Caucasian
17	Carrier (obligate)	50.7	Carrier	c.1274_1277dupTATC	Ashkenazi Jewish
18	Carrier (obligate)	35.6	Carrier	c.1274_1277dupTATC	Ashkenazi Jewish
19	Carrier (obligate)	35.3	Carrier	c.1274_1277dupTATC	Mediterranean/Irish/English/Slovakian
20	Carrier (obligate)	48.1	Carrier	c.1274_1277dupTATC	Ashkenazi Jewish/Caucasian
21	Carrier (obligate)	39	Carrier	c.1274_1277dupTATC	Ashkenazi Jewish
22	Carrier (obligate)	39	Carrier	c.1274_1277dupTATC	Ashkenazi Jewish
23	Carrier (obligate)	47.4	Carrier	c.1274_1277dupTATC	Irish/English/Czechoslovakian
24	Carrier (obligate)	29.4	Inconclusive (High Total HexA)	c.1274_1277dupTATC	Ashkenazi Jewish
25	Carrier	50.4	Carrier	c.1073+1G>A	Hispanic/French Canadian/German
26	Carrier	41.4	Carrier	c.1073+1G>A	Caucasian
27	Not reported	48	Carrier	c.1073+1G>A	Caucasian
28	Carrier (obligate)	46.2	Carrier	c.1073+1G>A	Caucasian
29	Carrier (obligate)	46.5	Carrier	c.1073+1G>A	Irish/Caucasian
30	Carrier (obligate)	44.4	Carrier	c.1073+1G>A	Caucasian
31	Carrier (obligate)	39.9	Carrier	c.1073+1G>A	Italian/Irish
32	Carrier (obligate)	49.1	Carrier	c.1073+1G>A	Irish
33	Carrier (obligate)	48.7	Carrier	c.1421+1G>C	Ashkenazi Jewish
34	Carrier (obligate)	36.6	Carrier	c.1421+1G>C	Ashkenazi Jewish
35	Carrier (obligate)	46.4	Carrier	c.1421+1G>C	Ashkenazi Jewish/Caucasian
36	Carrier (obligate)	34.6	Carrier	c.1421+1G>C	Ashkenazi Jewish
37	Carrier	42.3	Carrier	c.1393G>A (p.Asp465Asn)	Caucasian
38	Carrier (obligate)	50.8	Carrier	c.1393G>A (p.Asp465Asn)	Caucasian
39	Carrier (obligate)	45.7	Carrier	c.1393G>A (p.Asp465Asn)	Caucasian
40	Carrier (obligate)	44.3	Carrier	c.1510C>T (p.Arg504Cys)	Dutch/English

41	Carrier (obligate)	49.4	Carrier	c.1510C>T (p.Arg504Cys)	Caucasian
42	Carrier (obligate)	48.8	Carrier	c.965A>T (p.Asp322Val)	French Canadian/Irish
43	Carrier (obligate)	31.6	Carrier	c.965A>T (p.Asp322Val)	French Canadian/Irish
44	Carrier	44.6	Carrier	c.805G>A (p.Gly269Ser)	Slovakian/Polish/Eastern European
45	Carrier (obligate)	45.8	Carrier	c.805G>A (p.Gly269Ser)	Ashkenazi Jewish
46	Carrier	47.8	Carrier	c.775A>G (p.Thr259Ala)	Caucasian
47	Carrier (obligate)	48	Carrier	c.775A>G (p.Thr259Ala)	Caucasian
48	Carrier (obligate)	62.4	Non-carrier	c.533G>A (p.Arg178His)	Italian/Polish
49	Carrier (obligate)	60.9	Non-carrier	c.533G>A (p.Arg178His)	Hispanic
50	Carrier (obligate)	32.2	Carrier	del 7.6kb	French Canadian
51	Carrier (obligate)	66.8	Inconclusive (Low Total HexA)	c.1496G>A (p.Arg499His)	Irish/Caucasian
52	Carrier (obligate)	46.5	Carrier	c.927_928delCT	Mediterranean/Irish/Swedish
53	Carrier (pseudodeficiency allele)	51	Carrier	c.739C>T (p.Arg247Trp)	Ashkenazi Jewish
54	Carrier (obligate)	32.4	Inconclusive (High Total HexA)	c.459+5G>A	Hispanic
55	Carrier (obligate)	52.8	Carrier	c.118delT	Irish/mixed European
56	Not reported	32.3	Carrier	c.110A>G (p.Tyr37Cys)	Polish
57	Non-carrier	39.2	Inconclusive (High Total HexA)	no mutation detected	Caucasian
58	Non-carrier	65	Non-carrier	no mutation detected	Caucasian
59	Non-carrier	64.6	Non-carrier	no mutation detected	Caucasian
60	Non-carrier	60.1	Non-carrier	no mutation detected	Ashkenazi Jewish
61	Non-carrier	65.5	Non-carrier	no mutation detected	Caucasian
62	Non-carrier	66.3	Non-carrier	no mutation detected	Hispanic
63	Non-carrier	73	Non-carrier	no mutation detected	Ashkenazi Jewish/Caucasian
64	Non-carrier	64.6	Non-carrier	no mutation detected	Caucasian
65	Not reported	58.2	Inconclusive	no mutation detected	Ashkenazi Jewish
66	Not reported	53.2	Inconclusive	no mutation detected	Indian/French Canadian/Caucasian
67	Not reported	57.4	Inconclusive	no mutation detected	French Canadian/Caucasian
68	Not reported	62	Non-carrier	no mutation detected	Caucasian
69	Not reported	61.4	Non-carrier	no mutation detected	Africian American/Native American
70	Not reported	60.5	Non-carrier	no mutation detected	Africian American
71	Not reported	69.5	Non-carrier	no mutation detected	Native American/Caucasian
72	Not reported	61.1	Non-carrier	no mutation detected	Ashkenazi Jewish
73	Sandhoff	74.4	Non-carrier (Sandhoff)	no mutation detected	Caucasian
74	Sandhoff	80.7	Non-carrier (Sandhoff)	no mutation detected	French Canadian/Irish/Caucasian

Participants with HexA enzymatic activity in the ~36-52% range were classified as carriers, while those in the ~60-73% range were considered to be non-carriers, and those in the ~76-85% range were classified as Sandhoff (evaluated in the context of the total Hex activity as well). HGVS-approved variant nomenclature was used. GenBank accession number NM_000520.4.
